# In Vivo Evaluation of an Antibody-Functionalized Lipoidal Nanosystem for Schistosomiasis Intervention

**DOI:** 10.3390/pharmaceutics14081531

**Published:** 2022-07-22

**Authors:** Tayo A. Adekiya, Pradeep Kumar, Pierre P. D. Kondiah, Yahya E. Choonara

**Affiliations:** Wits Advanced Drug Delivery Platform Research Unit, Department of Pharmacy and Pharmacology, School of Therapeutic Sciences, Faculty of Health Sciences, University of the Witwatersrand, Johannesburg, 7 York Road, Parktown 2193, South Africa; adekiyatalex@gmail.com (T.A.A.); pradeep.kumar@wits.ac.za (P.K.); pierre.kondiah@wits.ac.za (P.P.D.K.)

**Keywords:** praziquantel, *Schistosoma mansoni*, nanoliposomes, anti-calpain, antibody, surface-functionalization

## Abstract

This study employed nanotechnological techniques to design and develop a praziquantel nanoliposomal (NLP) system and surface-functionalized the NLP with anti-calpain antibody (anti-calpain-NLP) for targeted praziquantel (PZQ) delivery in the treatment of schistosomiasis. Anti-calpain-NLPs were prepared and validated for their physicochemical parameters, in vitro and in vivo toxicity, drug entrapment efficiency (DEE), drug loading capacity (DLC), drug release, and parasitological cure rate. The particle sizes for the formulated nanoliposomes ranged from 88.3 to 92.7 nm (PDI = 0.17–0.35), and zeta potential ranged from −20.2 to −31.9 mV. The DLC and DEE ranged from 9.03 to 14.16 and 92.07 to 94.63, respectively. The functionalization of the nanoliposome surface was stable, uniform, and spherical. Fourier-transform infrared (FTIR), thermal behavior and X-ray powder diffraction (XRPD) analysis confirmed that the anti-calpain antibody and PZQ were attached to the surface and the nanoliposomes inner core, respectively. The drug sustained release was shown to be 93.2 and 91.1% within 24 h for NLP and anti-calpain-NLP, respectively. In the in vitro analysis study, the nanoliposome concentrations range of 30 to 120 μg/mL employed revealed acceptable levels of cell viability, with no significant cytotoxic effects on RAW 264.7 murine macrophage as well as 3T3 human fibroblast cells. Biochemical markers and histopathological analysis showed that the formulated nanoliposomes present no or minimal oxidative stress and confer hepatoprotective effects on the animals. **The cure rate of the anti-calpain-NLP and PZQ was assessed by parasitological analysis, and it was discovered that treatment with 250 mg/kg anti-calpain-NLP demonstrated greater activity on the total worm burden, and ova count for both the juvenile and adult schistosomes in the intestine and liver of infected mice.** The findings so obtained supported the ability of oral anti-calpain-NLP to target young and adult schistosomes in the liver and porto-mesenteric locations, resulting in improved effectiveness of PZQ.

## 1. Introduction

Neglected tropical diseases (NTDs) are groups of disabling, chronic, and disfiguring diseases that have been abandoned in favor of cancer, diabetes, tuberculosis, malaria, and other well-known diseases because they are most prevalent in poverty extreme areas, particularly among some disadvantaged urban and the rural poor populations. Meanwhile, among the NTDs, the second most common is schistosomiasis caused by *Schistosoma* parasites [1,2]. Approximately, 800 million people are currently living with this disease globally, with over 200,000 deaths yearly with sub-Saharan Africa with the highest proportion of this population [3]. *S. mansoni* (intestinal) and *S. haematobium* (urogenital) are the most prevalent and cause the highest disease burden in Africa, which account for approximately 90% in sub-Saharan Africa [4,5]. The full impact of the disease holds a major effect on the government and households, health, financial, social, and economic conditions. Anemia, fever, genital lesions, stunting, and permanent organ damage are some symptoms of schistosomiasis [5]. Clinical reports of the schistosomiasis of the cervix, which emerged in the mid-20th century, are based on comprehensive female genital schistosomiasis (FGS) descriptions based on microscopy of genital biopsies and colposcopic examination confirmed *S. haematobium* eggs in the vagina, cervix, and vulva [6]. FGS is a well-known outcome of *S. haematobium* parasitism, affecting around half of the infected females (33 to 75%), or approximately 40 million girls and women. As a result, it is one of Africa’s most frequent gynecologic conditions [6]. Additionally, several studies have reported seizures and cerebral schistosomiasis in different *Schistosoma* spp. Case reports using MRI, PCR, and computed tomography (CT) analyses [7,8,9,10,11,12,13].

The major drug and standard treatment for schistosomiasis are PZQ, due to its efficacy against all adult forms of *Schistosoma* species and has been well-tolerated by the affected populations. It also decreases parasite load and symptoms severity [14]. Thus, the use of PZQ has some significant problems which are: inefficiency against the immature Schistosoma species, and after intake, there is a fast absorption into the circulation and significant first-pass metabolism [15,16]. other problems include less bioavailability, less solubility, and multidrug-resistant strains, which have been reported in an endemic area as well as described in the laboratory [17,18]. It is obvious that the mass PZQ-based method must be thoroughly investigated in terms of long-term sustainability and efficacy [4]. However, the design and development of new drugs for this disease have been faced with several shortcomings, such as insufficient financial support and motivation from pharmaceutical companies, as well as a huge cost associated with the process of new chemical entities (NCEs) due to the long drug discovery pipeline and lack of interest from researchers in the area of NTDs [18,19]. Hence, in the absence of a vaccine [4], our current study uses a nanomedicine approach to optimize the existing anti-schistosomal gold standard drug, PZQ.

Nanomedicine, which uses nanotechnology as a tool for monitoring, treatment, control, and prevention of biological diseases, has been a promising approach to improving pharmaceutical ingredients in the treatments of several diseases and disorders [18]. Thus, nanoliposomes, which are vesicles comprising concentric bilayer phospholipids that may encapsulate or entrap hydrophobic or hydrophilic drugs and deliver them to specific locations in the body through a targeted drug delivery approach for the treatment of diseases, have been utilized [20]. Lipid-based formulations have gained a lot of attention for improving drug absorption and bioavailability and, more importantly, reducing some of the adverse effects [19]. Furthermore, due to their amphipathic nature, nanoliposomes have attracted substantial attention in drug delivery, making them important for the enhancement and improvement in the absorption of drugs across biological barriers at the rate at which drugs may be released and in the solubilized form [21]. On the other hand, nanoliposomes can reduce blood circulation times due to significant absorption by the reticuloendothelial system (RES) macrophage cells. Several studies have revealed that PZQ-loaded liposomes improved the anti-schistosomal activity of PZQ, and this can serve as a base for alternative oral nanocarrier for drug administration. Furthermore, the European Medicines Agency (EMA) and the Food and Drug Administration (FDA) of the USA have endorsed numerous liposomal formulations for clinical usage.

Liposomes with modified surfaces containing polyethylene glycol (PEG), silk-fibroin, chitosan, or polyvinyl alcohol (PVA) have been found to increase the half-life liposome blood circulation. However, several studies have employed a similar approach with positive outcomes such as gold nanoparticles conjugated with antibodies as tumor-targeting radiosensitizers for proton therapy, and a potential strategy to improve cell killing [22]. The functionalization of multilayered particles with monoclonal antibodies for cancer cell targeting is capable of preserving the protein coronas, an essential protein that determines the surface characteristics of particles and their targeting capabilities [23]. Other positive outcomes include the drug-conjugated aptamer for the doxorubicin-targeted delivery into HER3 cell for cardiotoxicity reduction in breast cancer treatment [24], the functionalization of polymeric nanoparticles with mannose for therapeutic anti-tumor immune responses, and the induction of prophylactic in a melanoma model [25].

Thus, this study would be the first to design a nanoliposome surface-engineered with an antibody (anti-calpain) for the PZQ-targeted delivery in schistosomiasis treatment. There are several target proteins and molecular receptors located on the schistosome tegument surface, such as schistosome cysteine protease calpain, which belongs to the calpain family that is upregulated in the Schistosoma tegument [26,27]. Schistosome glucose transporter 1 (SGTP1) and 4 (SGTP4) are found in all types of schistosomes [28,29]. A nicotinic form of acetylcholine receptor (nAChR) and acetylcholinesterase (AChE) is mostly present on the surface of male schistosomes [30,31]. Another targeted protein found on the tegument surface is dynein (*Schistosoma mansoni*) [31]. For this study, cysteine protease calpain was the targeted protein on the schistosomes because the activities of calpain occur at the sites of extracellular cell–cell adhesion and cell–substrate adhesion. In schistosomes, calpain is part of the expressed tegumental proteases, which are upregulated at the host–parasite interface. They are located in the cytoplasmic extensions of microtubular bundles that connect the surface syncytial epithelium to the schistosomes cell bodies underlying the muscle of both the juvenile and adult schistosomes [26,27]. In that location, they perform similar functions with other calpains, such as the transportation and delivery of membrane precursors, that is, as a mediator in the synthesis of surface membranes. They are also found across the syncytial layer, where they function with the apical plasma membrane, causing membrane turnover [26]. Based on the function of calpains in the mediation of calcium signaling processes, biogenesis of surface membrane, and immunological evasion, they have been proposed as a potential candidate for chemotherapy targets and/or for a define-molecular vaccine [26,27].

Thus, this study employed nanotechnological techniques to design and develop a praziquantel nanoliposomal (NLP) system and surface-functionalized the NLP with anti-calpain antibody (anti-calpain-NLP) for targeted praziquantel (PZQ) delivery in the treatment of schistosomiasis. The NLP designed in this study has a significant effect on targeting adult and juvenile schistosomes and improves the specificity and efficacy in cells and tissues through overexpressed surface receptors (cysteine protease calpain). Additionally, the lipoidal nanosystem could clear all the traces of drug resistance and avert the reinfection of the disease by scavenging all the remnants of the parasite in the human host body.

## 2. Materials and Methods

### 2.1. Materials

Praziquantel, phospholipids (Asolectin from soybean), cholesterol (CHOL), phosphatidylethanolamine distearoyl methoxypolyethleneglycol conjugate (DSPE-mPEG^2000^COOH), N-hydroxysulfosuccinimide (NHS), *N,N*′ -dicyclohexylcarbodiimide (DCC), and 49,409 Atto 488 Phalloidin and Biochemical assays (AST, ALT, ALP, bilirubin, and creatinine) kits were purchased from Sigma-Aldrich (St. Louis, MO, USA). DAPI stain was purchased from Thermofisher. Monoclonal calpain antibody was purchased from Abcam (ab154167; Cambridge, UK) raised from rabbit; RAW 264.7 murine macrophage and 3T3 human fibroblast cell lines were procured from ATCC (Manassas, VA, USA). All other chemicals used in the study were of analytical grade.

### 2.2. Methods

#### 2.2.1. Nanoliposome Preparation

Nanoliposomes (NL) were formulated by adapting the reverse-phase evaporation method formulated by Mufamadi et al. [20]. Briefly, the mixture of methanol and chloroform (1:9 *v*/*v*) was used to dissolve asolectin, CHOL, and mPEG^2000^COOH, and these were mixed with a probe sonicator in a round-bottom flask for 30 s, at 60 rpm. Thereafter, a rotavap maintained at 60–65 °C for 3 h was used to evaporate the solvents, to obtain a lipid thin film on the flask wall. An adequate amount of preheated phosphate-buffered saline (PBS) at 60 °C was added thereafter and the vessel was agitated vigorously on a rotary mixer to form multilamellar vesicles (MLVs).

To generate the PZQ-loaded nanoliposome (NLP), PZQ was dissolved in a methanol–chloroform solution containing NL in a molar ratio of 1:6 for PZQ/NL, and this was hydrated in 4 mL of pH 7.4 PBS buffer, at 65 °C, for 30 min. Subsequently, the mixture was dialyzed against deionized water to eliminate the ammonia and residual drug after it had been cooled to 4 °C. Unilamellar nanoliposomes were generated through a freeze–thaw method. Briefly, the solutions of nanoliposome were frozen at −80 °C by liquid nitrogen and thereafter thawed in a water bath set at 37 °C (*n* = 6) [32]. Moreover, samples were put on an ultrasonic bath for 5 min at 50 kHz before being stored at 4 °C. Slow and steady extrusion of the nanoliposome formulation via a polycarbonate membrane filter of μm pore size was used to generate fine and uniform particle size.

#### 2.2.2. Surface-Engineering of the Nanoliposomes with Anti-Calpain

The monoclonal calpain antibody from Abcam (ab124631; Cambridge, UK) was purchased. The anti-calpain-functionalized PZQ-loaded nanoliposomes were briefly synthesized following the use of the technique described by Mufamadi et al. [20], by reacting native nanoliposomes with 87 mg of DCC dissolved in DMSO (100 μL) in the presence of 46 mg of NHS in 4 mL of PBS (pH 7.2). Then, 10 μL of 50 μg (0.68 mg/mL) of the anti-calpain was mixed with the treated suspension of nanoliposome and allowed to mix for about 6 h at room temperature. Subsequently, solvents were precipitated by a rotary evaporator for 2–3 h at 65 °C in a water bath. This was followed by the dialysis of anti-calpain-functionalized PZQ-loaded nanoliposomes (anti-calpain-NLP) using SnakeSkin pleated dialysis tubing for about 24 h to eliminate excess NHS, DCC, and uncoupled antibodies in DDW. The anti-calpain-functionalized nanoliposome was stabilized through the use of a freeze–thaw process and stored at 4 °C for future use. Thereafter, the antibody coupling efficiency (ACE) on the surface of the nanoliposomes was measured using a NanoPhotometer spectrophotometer at 260 nm wavelength against the normal nanoliposomes. This was achieved by the addition of 2 mL of 0.5% (*v*/*v*) triton X-100 in methanol with 2 mL of the antibody-functionalized nanoliposome solution, then by allowing it to react for 2 to 3 h, at 45 °C. The ACE value validated the total quantity of antibodies that was attached to the surface of the nanoliposomes, and it was calculated using the following equation:

(1) $$\mathrm{ACE}\%=\;\frac{A_{q}}{T_{q}}\;\times \;100$$

where *A_q_* is the actual amount of antibody attached to the surface of the nanoliposome and *T_q_* is the theoretical amount of antibody employed during the coupling procedure to synthesize the antibody-functionalized nanoliposomes.

#### 2.2.3. Morphological Analysis

##### Scanning Electron Microscopy

The morphological surface of the anti-calpain-NLP was determined by employing SEM analysis (SIGMA VP, Zeiss Electron Microscopy, Carl Zeiss Microscopy Ltd.; Cambridge, UK). A drop of NLP suspensions was placed on a metallic sample stub and left overnight to dry. Subsequently, the samples loaded on metallic sample stubs were sputter-coated with both gold and palladium and covered with a carbon layer to reduce the surface charging. Thereafter, each sample was observed under different magnifications at an accelerated voltage of 20 kV.

##### Transmission Electron Microscopy

Transmission electron microscopy (TEM) version TECNAIF3OST-TEM was employed in ascertaining the elemental distribution of the anti-calpain-NLP and the morphology of the particles. Dispersions were concentrated in PBS buffer at pH 7.4; in a ratio of about 1:10 and ultrasonicated for 5 min, at 37 °C. A drop of the concentrated sample was mounted for 5 min on a carbon-coated copper grid following the excess removal of liquid; this was attained through blotting with filter paper and then air-dried at 25 °C. Subsequently, the films on the copper grid were visualized under an electron microscope at 10,000× magnifications.

#### 2.2.4. Particle Size Distribution, Zeta Potential, and Polydispersity Index (PDI) Analysis

The polydispersity index (PDI), average particle size, and zeta potential of NL, NLP and PZQ-loaded anti-calpain-functionalized nanoliposomes were measured by a Malvern ZetaSizer Nano ZS (Malvern Instruments, Worcestershire, UK), at 25 °C. All nanoliposomes’ PDI, zeta potential, and particle size measurements were performed following the same method, that is, each sample was concentrated (1:10) with deionized water for each run by disposable cuvettes. Each test was carried out in triplicate, and the mean value in each case was documented accordingly.

#### 2.2.5. Evaluation of the Drug Entrapment Efficiency of the PZQ-Loaded Nanoliposomes

To ascertain the entrapment efficiency of PZQ inside the nanoliposomes, 2 mL of PZQ-nanoliposomes suspension (*n* = 3) was centrifuged for 1 h, at 5000 rpm. The amount of drug in the clear supernatant was analyzed at a wavelength of λmax = 265 nm using UV-Vis spectroscopy (Specord40, Analytik Jena, AG, Jena, Germany) and calculated using a PZQ standard linear curve in concentrations range of 0 and 16 μg/mL in double-distilled water (R^2^ = 0.99). Triplicate measurements were determined to ascertain the percentage value of EE and drug loading capacity of the nanoliposomes by Equations (2) and (3) below, respectively;

(2) $$\%\mathrm{EE}=\frac{\mathrm{Total}\;\mathrm{amount}\;\mathrm{of}\;\mathrm{PZQ}\;\mathrm{loaded}\;-\;\mathrm{Amount}\;\mathrm{of}\;\mathrm{PZQ}\;\mathrm{in}\;\mathrm{the}\;\mathrm{supernatant}}{\mathrm{Total}\;\mathrm{amount}\;\mathrm{of}\;\mathrm{PZQ}\;\mathrm{loaded}}\;\times \;100$$

(3) $$\%\mathrm{LC}=\;\frac{\mathrm{Amount}\;\mathrm{of}\;\mathrm{PZQ}\;\mathrm{in}\;\mathrm{nanoliposomes}}{\mathrm{The}\;\mathrm{weight}\;\mathrm{of}\;\mathrm{nanoliposomes}}\;\times \;100$$

#### 2.2.6. Fourier-Transform Infrared Spectroscopy (FT-IR)

Fourier-transform infrared (FTIR) analysis of the PZQ, NL, NLP, and anti-calpain-NLP was determined to ascertain the characteristics of the possible interactions of the molecular structure of the antibody and the nanoliposomes consisting of mPEG^2000^COOH on the nanoliposomes surface. The analysis was determined using Perkin-Elmer spectrum 2000 ATR-FTIR (PerkinElmer 100, Llantrisant, Wales, UK) via the KBr pellets method, with 4 cm^−1^ resolution in the region of 4000–650 cm^−1^.

#### 2.2.7. X-ray Powder Diffraction (XRPD) Analysis

The degree of crystallinity transitions of the PZQ, NL, NLP, and anti-calpain-NLP in lyophilized form were investigated utilizing x-ray powder diffraction (XRPD) spectra (Rigaku MiniFlex 600, Tokyo, Japan) at 40 kV and 15 mA sourced with CuKα radiation. The 2θ scan ranging from 3° to 90° was chosen, and a scanning rate of 10° per minute was used to achieve diffractograms of the samples.

#### 2.2.8. Differential Scanning Calorimetry (DSC) Analysis

The thermal attributes of the PZQ, NL, NLP, and anti-calpain-NLP in lyophilized form were analyzed on a DSC by a Mettler Stare system provided with STARe SW software. This physicochemical parameter was achieved by weighing 3 to 10 mg depending on each sample in a pin-hole alumina crucible and heated under a nitrogen flow, from 30 °C to 300 °C, with a heating rate of 5 °C/min.

#### 2.2.9. Thermogravimetric Analyzer (TGA) Analysis

The range of thermal degradation of the PZQ, NL, NLP, and anti-calpain-NLP was evaluated by employing a thermogravimetric analyzer (PerkinElmer, TGA 4000, Llantrisant, Wales, UK). The samples were heated up to 900 °C, from 30 °C, with a 10 °C/min heating rate under the continuous flow of nitrogen. Thermograms generated were given in percentage weight against temperature.

#### 2.2.10. In Vitro Release of the Drugs

PZQ release from PZQ-nanoliposomes dispersion was determined in a dissolution medium containing 100 mL of PBS (pH 7.4) consisting of Tween 80 (0.002%) through a dialysis technique in a thermostatically controlled horizontal orbital shaking incubator (type LM-530, Yihder Technology Co., LTD., New Taipei City, Taiwan) set at 25 rpm, for 24 h, at 37 ± 1 °C. The PZQ suspension (25 mg/mL) was utilized as the control. At given time intervals (1, 2, 4, 8, 12, and 24 h), 2 mL of the samples was drawn for analysis and an equivalent amount of pre-warmed fresh media was replaced into the samples to sustain sink conditions. The samples withdrawn were analyzed for drug (PZQ) release using a nanophotometer at 263 nm. The amount of PZQ released was calculated from a PZQ standard linear curve (R^2^ = 0.99) in PBS (pH 7.4). All experiments were carried out in triplicate. The values of mean dissolution time (MDT) was calculated for each sample utilizing Equation below:

(4) $$MDT=\overset{n}{\underset{i=1}{\sum }}\mathrm{ti}\left(\frac{M_{t}}{M_{\infty }}\right)\;$$

where the fraction of amount released in time *ti = (ti + ti −* 1)/2 denotes *M_t_*, and *M*_∞_ represents the ejecting amount.

#### 2.2.11. In Vitro Cytotoxicity Assay (MTT Assay)

The in vitro cytotoxicity of the PZQ, NL, NLP, and anti-calpain-NLP in RAW 264.7 murine macrophage cell and 3T3 human fibroblast cell was investigated by an MTT cell viability assay. The cells were cultured in DMEM and RPMI, respectively, supplemented with 1% penicillin-streptomycin and 10% fetal bovine serum in a humidified incubator at 37 °C and 5% CO_2_. Every two days, fresh media were used to replace the growth medium until the cells reached about 80 to 90% confluence. In a 96-well plate, the cells were seeded at a density of 1 × 10^5^ cells per well. After 24 h, the adhered cells were treated with NLP, anti-calpain-NLP, and PZQ at a range concentration range of 30 to 120 μg/mL, undertaken in triplicate. Subsequently, the cells were incubated in a humidified incubator for 24 h at 5% CO_2_ and 37 °C. After 24 h, 10 μL of MTT reagent (Merck, Darmstadt, Germany) was added to the wells, and the cells were incubated for 4 h, at 37 °C. Thereafter, 110 μL of DMSO was added, and the well plates were further incubated for an hour, at 37 °C, to dissolve the formazan crystals. Thereafter, the absorbance was calculated at 570 nm, with a reference wavelength of 690 nm using a multimode microplate reader (FilterMaxTM F5, Molecular Devices, CA, USA). The ratio of absorbance of the test samples to that of the untreated samples was used to compute the percentage of cell viability (control).

(5) $$\mathrm{Percentage}\;\mathrm{cell}\;\mathrm{viability}=\frac{\left(\mathrm{Absorbance}\;\mathrm{of}\;\mathrm{the}\;\mathrm{test}\;\mathrm{sample}\right)\times 100}{\left(\mathrm{Absorbance}\;\mathrm{of}\;\mathrm{untreated}\;\mathrm{sample}\;\left(\mathrm{control}\right)\right)}$$

#### 2.2.12. Cell Morphology Analysis

As described in the previous section, after RAW 264.7 murine macrophage cells cultured reached about 80 to 90% confluence, the cell was seeded at a density of 2.5 × 10^6^ cells/mL and cultured on coverslips in 6-well plates containing 2 mL DMEM supplemented with 10% (*v*/*v*) FBS and 1.0% (*v*/*v*) penicillin–streptomycin antibiotic and incubated for 24 h, at 37 °C, under 5% CO_2_. After 24 h, the adhered cells were treated with 30 to 120 μg/mL concentration of NL, NLP, anti-calpain-NLP, and PZQ as well as 10 μg/mL of 5-fluorouracil (5-FU) as a negative control in triplicate. Subsequently, the cells were further incubated for 24 h, at 37 °C and 5% CO_2_, in a humidified incubator. Upon 24 h of treatment, the phase-contrast morphology of the cells was visualized under an inverted compound light microscope (Olympus CKX53, Olympus Corporation, Tokyo, Japan). Thereafter, the cells were fixed by first spiking the media growth containing the cells with 500 μL of 4% formaldehyde to avoid damaging the cells by the abrupt change between the fixation solution osmolarity and culture medium osmolarity. Then, the media were aspirated and decanted after 2 min, and subsequently, the cells were covered with fresh 1 mL of 4% formaldehyde for about 20 min. After 20 min of fixation, the fixed cells were washed gently with 2 mL of PBS 4 times to remove any unbound fixation agent; thereafter, the cells were stained with 500 μL of a 50 μM fluorescent phalloidin solution for 40 min, at room temperature, under dark conditions. Then, 40 min later, the cells were washed 4 times with PBS to remove unbound phalloidin stain; subsequently, the cells were stained with 500 μL of 500 nM DAPI stain solution and incubated for 5 min, at room temperature, in the absence of light. Afterward, the cells were rinsed thoroughly with PBS to remove the unbound DAPI stain solution. Then, the coverslips were viewed after being mounted on glass slides under the compound fluorescent microscope (Olympus IX51, Olympus Corporation, Tokyo, Japan).

#### 2.2.13. In Vivo Toxicity

The evaluation of in vivo toxicity was performed in Sprague Dawley rats, with a weight range of 250–300 g, kept in a standard husbandry and housing condition, at normal room temperature, and fed with normal tap water and normal rat chow. The animals were allocated randomly to four (4) groups: Group I: control; Group II: PZQ; Group III: PZQ-NLP; Group IV: PZQ-loaded anti-SGTP4-functionalized NLP. An equivalent amount of the dose (250 mg/kg) in about 2 to 3 mL of PZQ, PZQ-NLP, and PZQ-loaded anti-SGTP4-functionalized NLP was administered orally to the animals via the once-off oral gavage. Seven days later, all the animals were euthanized, and their blood was taken via cardiac puncture and stored in microcentrifuge heparin tubes, and centrifuged for 5 min at 2500 rpm, at 4 °C, to isolate plasma in red blood cells (RBCs). Thereafter, the plasma was examined for a liver functioning test to assess the hepatotoxicity of the various formulations by the measurement of the plasma levels of aspartate aminotransferase (AST), alanine aminotransferase (ALT), creatinine and bilirubin using commercially available kits from Sigma-Aldrich, Johannesburg, Gauteng, South Africa. The activities of ALT, AST, creatinine, and bilirubin were calculated for each assay using Equations below;

(6) $$\mathrm{ALT}\;\mathrm{activity}=\frac{B\times \mathrm{Sample}\;\mathrm{Dilution}\;\mathrm{Factor}}{\left(\mathrm{Final}\;\mathrm{temperate}-\mathrm{Initial}\;\mathrm{temperature}\right)\times V\;}$$

where *B* is the pyruvate amount (nmole) generated between the initial temperature and final temperate, and the final temperature is the time of first reading in minutes, while the initial temperature is the penultimate time reading in minutes, and *V* is the volume (mL) of the sample added to well.

(7) $$\mathrm{AST}\;\mathrm{activity}=\frac{B\times \mathrm{Sample}\;\mathrm{Dilution}\;\mathrm{Factor}}{\left(\mathrm{Reaction}\;\mathrm{Time}\right)\times V\;}$$

where *B* is the amount (nmole) of glutamate produced between the initial temperature and final temperate, the reaction time is the final temperature minus the initial temperature in minutes and *V* is the volume (mL) of sample added to the well.

(8) $$\mathrm{Concentration}\;\mathrm{of}\;\mathrm{creatinine}=\frac{\mathrm{Sa}}{\mathrm{Sv}}$$

where *Sa* is the creatinine amount (nmole) in the unknown sample from the standard curve and *Sv* is the volume (μL) of sample added into the wells, while C is the creatinine concentration in the sample and the molecular weight of creatinine is taken as 113.12 g/mole.

(9) $$\mathrm{Concentration}\;\mathrm{Bilirubin}=\frac{(A_{530})\mathrm{sample}-(A_{530})\mathrm{blank}}{(A_{530})\mathrm{calibrator}-(A_{530})\mathrm{water}}\times \left(5\;mg/dL\right)$$

where (*A*_530_) sample is the value of the sample (direct or total) and (*A*_530_) blank is the sample blank value, while (*A*_530_) calibrator and (*A*_530_) water are the calibrator reading value and the water control reading value, respectively. The amount of 5 mg/dL is the equivalent bilirubin concentration of the calibrator using a multimode microplate reader (FilterMaxTM F5, Molecular Devices, CA, USA).

#### 2.2.14. Sample Preparation for Histopathological Analysis

To determine the toxicity of the nanoliposome formulations to the organ, tissue samples (liver, lung, kidney, and spleen) were obtained and placed in 10% neutral buffered formalin, thereafter inserting them in paraffin for sectioning the tissues. Then, the tissues were stained with eosin and hematoxylin for microscopic determination and spotted for toxicity markings according to IDEXX protocol (IDEXX-JB632262).

#### 2.2.15. In Vivo Antischistosomal Study

##### Infection of Animals

The in vivo antischistosomal study was carried out in the Theodor Bilharz Research Institute (TBRI), Schistosome Biological Supply Centre (SBSC) section, Giza, Egypt. Male Swiss albino mice (CD-1) weighing 18–20 g were obtained from SBSC of TBRI, Giza, Egypt, and housed in a controlled environment, at a room temperature of 20–22 °C, a 12 h light/dark cycle, and 50–60% humidity all through the acclimatization and experimental periods, with access to food and water ad libitum. The cercarial suspension (0.1 mL) was gently mixed, stained with a picric acid solution, and counted.

Subsequently, the mice were subcutaneously infected with *S. mansoni* cercariae (supplied by SBSC) and exposed to 60 ± 10 cercariae/mouse. All animal studies were carried out in compliance with the TBRI Institutional Review Board’s Guide for the Care and Use of Laboratory Animals.

##### Experimental Design and Animals Grouping

Mice infection: *S. mansoni* cercariae were inoculated and infected subcutaneously with 60 ± 10 strain of Egyptian *S*. *mansoni* cercariae sheds from *Biomphalaria alexandrina* snail in accordance to Mossallam et al. [33].

Animals were divided according to the time of drug administration:

**Group 1**: Infected control;
**Group 2**: Single dose of 250 mg/kg of Anti-calpain-PZQ-loaded nanoliposomes was given two weeks post infection;
**Group 3**: A drug (PZQ) 250 mg/kg single dose was given two weeks post infection;
**Group 4**: Single dose of 250 mg/kg of Anti-calpain-PZQ-loaded nanoliposomes was given four weeks post infection;
**Group 5**: A drug (PZQ) 250 mg/kg single dose was given four weeks post infection.

All animals were sacrificed six weeks post infection.

##### Assessment of Parasitological Criteria of Cure

**Worm recovery**: Sacrificed mice were subjected to a hepatic-porto-mesentric perfusion procedure to collect adult *S. mansoni*, assess sex (male/female/copula), estimate worm load, and then compute the percentage of overall worm reduction [34].

**Oogram pattern**: The percentage of eggs at various stages of development (oogram pattern) was investigated [33,34]. The eggs were identified and counted at different developmental stages in three intestinal fragments, and the average amount of each stage was computed.

**Egg count in tissues**: Small sections of intestinal and hepatic tissues were weighed, digested overnight in a 5 mL KOH 5% solution, and three samples (each 50 μL) of the digested tissue were examined microscopically to calculate the mean egg count [34]. Kloetzel’s formula was used to calculate the number of eggs per gram of tissue as well as the percentage reduction in total ova per gram of tissue [35].

#### 2.2.16. Statistical Analysis

The results are expressed as mean values and standard deviation (±SD), and the significance of the difference observed was calculated using a Student’s *t*-test on GraphPadprism version 9 (GraphPad Prism software, Inc., San Diego, CA, USA). In all the tests, *p*-values < 0.0001 are considered statistically significant.

Other data were coded and entered using the statistical packages Microsoft Excel 2016. The following calculation was used to compute the percentage reduction in worm/egg load in each treatment group: % reduction = [(No. of worms/eggs in the control group) − (No. of worms/eggs in the treated group)]/(No. of worms/eggs in the control group) × 100.

## 3. Results

### 3.1. Physicochemical Characterization of the Nanoliposomes

#### 3.1.1. Surface Morphology and Shape Analysis of the Formulated Nanoliposomes

**Figure 1:**
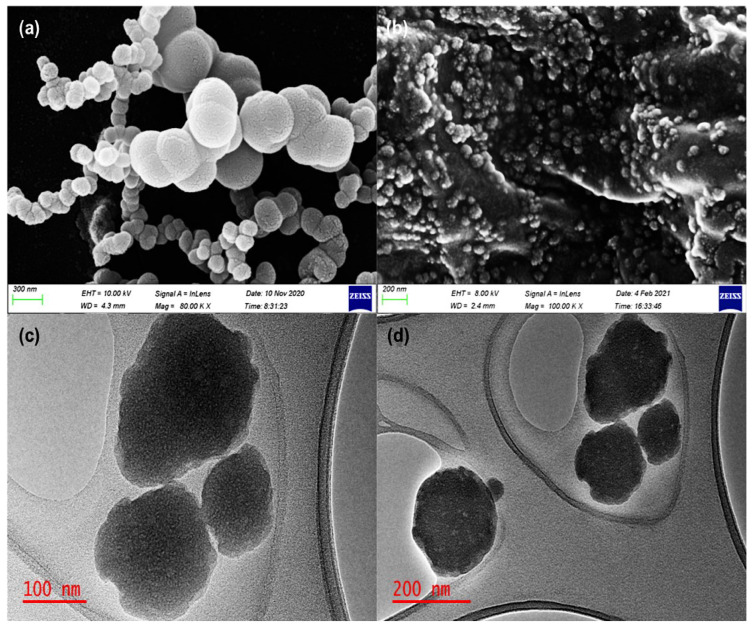
SEM images of NLP (**a**) and anti-calpain-NLP (**b**); TEM images of (**c**) anti-calpain-NLP at 100 nm and (**d**) at 200 nm.

Surface electron microscopy (SEM) and transmission electron microscopy (TEM) were used to evaluate the surface morphology and the shape of the formulated nanoliposomes. The SEM and TEM images in Figure 1a–d revealed that the formulated nanoliposomes are uniformly spherical in shape, with stable or intact structures, showing typical SEM and TEM images of the nanoliposomes. As further shown in Figure 1b, the SEM image of the anti-calpain-functionalized nanoliposomes presented a uniform surface morphology different from the unfunctionalized nanoliposomes shown in Figure 1a. These results also confirmed that the anti-calpain antibody was surface-conjugated onto the nanoliposomes. Interestingly, the morphology profile shown in Figure 1c,d for anti-calpain-NLP revealed that after the entrapment of PZQ inside the core and surface engineering of the antibody onto the nanoliposomes surface, there was no aggregation. Furthermore, the particle size distribution for anti-calpain-NLP was within the nanoscale, which corroborated the data obtained by the Malvern ZetaSizer Nano ZS.

#### 3.1.2. Particle Size Distribution, PDI, Zeta Potential, Drug Entrapment Efficacy, and Drug Loading Capacity Analysis

**Figure 2:**
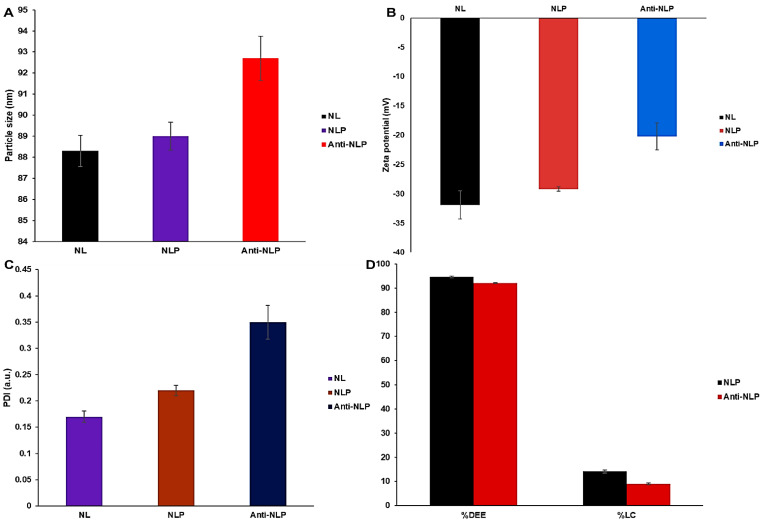
Particle size (**A**), zeta potential (**B**), PDI (**C**) of unloaded, PZQ-loaded, and Anti-calpain-PZQ-loaded nanoliposomes, and (**D**) the drug entrapment efficacy and drug loading capacity. PDI means polydispersity index, NL represents unloaded nanoliposomes, NLP means PZQ-loaded nanoliposomes, and AntiCal-NLP represents the Anti-calpain-engineered PZQ-loaded nanoliposomes. %DEE refers to drug entrapment efficacy, and %DLC refers to drug-loading capacity.

Figure 2 shows the particle sizes of the NL, NLP, and anti-calpain-NLP to be 88.3 ± 0.75, 89.0 ± 0.66, and 92.7 ± 2.85 nm, respectively, which showed that the formulated nanoliposomes fall within the good particle size range. Furthermore, the PDI for the nanoliposomes formulated were observed to be 0.17 ± 0.011, 0.22 ± 0.010, and 0.35 ± 0.032 a.u. for NL, NLP, and anti-calpain-NLP, respectively. This indicates that the nanoliposomes are stable regarding flocculation, aggregation, sedimentation, creaming, and coagulation through a strong electrostatic repulsion, which keeps the charges of the particles from one another. The zeta potential happens to be one of the significant parameters responsible for the stability of colloidal dispersions. Thus, the zeta potentials in this study are shown to be −31.9 ± 2.41, −29.2 ± 0.37, and −20.2 ± 2.28 mV for NL, NLP, and anti-calpain-NLP, respectively. These high zeta potential values conferred stability to all the nanoliposomes formulated and they resist aggregation of the particle dispersion.

Figure 2 further shows the drug entrapment efficacy and drug-loading capacity for the NLP and anti-calpain-NLP. This revealed that the nanoliposomes achieved a high PZQ entrapment efficacy of 94.63 ± 0.30 and 92.07 ± 0.23%, with a drug-loading capacity of 14.16 ± 0.61 and 9.03 ± 0.42%, respectively.

#### 3.1.3. Molecular Vibrational Transitions and X-ray Powder Diffraction of the Formulated Nanoliposomes Evaluation

**Figure 3:**
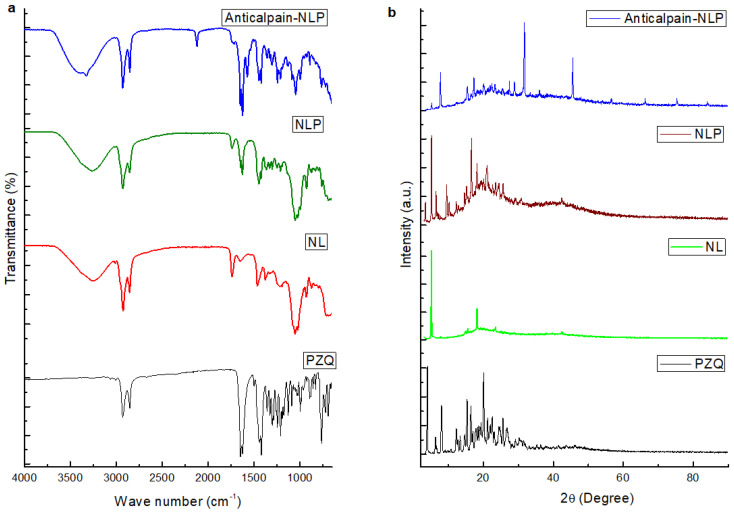
(**a**) FTIR spectra of PZQ, NL, NLP, and anti-calpain-NLP. The FTIR spectrum for the PZQ showed vibration peaks at the wavenumbers 2929.49 cm^−1^ and 2852.52 cm^−1^. NL displayed a broad FTIR spectrum band at wavenumber 3256 cm^−1^, and two bands were observed at wavenumbers 2924 cm^−1^ and 2853 cm^−1^. In NLP, the absorption peaks that corresponded to the peaks of PZQ disappeared, and the carboxyl group absorption at 1739 cm^−1^ stretched became weak and the peak absorption at 1640 cm^−1^ became stronger. Anti-calpain-NLP presented an absorption band at 1851 cm^−1^ and absorption at 1739 cm^−1^, while there is a slight shift and a very high peak absorption at 1624 cm^−1^. This indicates that amide(-NH_2_) bending vibrations are formed during the covalent binding of the anti-calpain antibody; (**b**) P-XRD images of PZQ, NL, NLP and anti-calpain-NLP.

The FTIR spectra of the PZQ, NL, NLP, and anti-calpain-NLP are illustrated in Figure 3a. The FTIR spectrum for the PZQ showed vibration peaks at the wavenumbers 2929.49 cm^−1^ and 2852.52 cm^−1^, which indicated the presence of symmetric CH and asymmetric CH_3_ stretching vibrations. The NL displayed a broad FTIR spectrum band at wavenumber 3256 cm^−1^, which could be ascribed to the absorption bands for the O-H; two bands were observed at wavenumbers 2924 cm^−1^ and 2853 cm^−1^, which could be attributed to -CH_2_ and -CH_3_ stretching vibrations. Furthermore, two bands were observed at 1739 cm^−1^ and 1640 cm^−1^, which are ascribed to the intensities of the carboxyl group vibration absorptions. Following the entrapment of PZQ with the nanoliposomes, the absorption peaks that corresponded to the peaks of PZQ disappeared, and the carboxyl group absorption at 1739 cm^−1^ stretched became weak and the peak absorption at 1640 cm^−1^ became stronger. This is an affirmation that there is an interaction between the PZQ and nanoliposomes during the process of drug entrapment. Anti-calpain-engineered PZQ-loaded nanoliposomes presented an absorption band at 1851 cm^−1^ and absorption at 1739 cm^−1^, while there is a slight shift and a very high peak absorption at 1624 cm^−1^. This indicates that amide(-NH_2_) bending vibrations are formed during the covalent binding of the anti-calpain antibody. FTIR analysis revealed an interaction between the antibody’s -NH2 group and the -OH group of DSPE-mPEG^2000^COOH during the formation of anti-calpain-engineered PZQ-loaded nanoliposomes.

The crystal nature of the PZQ, NL, NLP, and anti-calpain-NLP evaluated using powder X-ray diffraction technique are illustrated in Figure 3b. PZQ was observed to be crystallized in nature, with major peaks at 2θ-4.2°, 7.6°, 16.6°, 17.8°, and 20.3°; NL was shown to be crystalline, with specific peaks at 2θ-5.2° and 20.1°. Following the interaction of PZQ with NL, there is a slight shift in the peaks at 2θ-6.0° and 20.1°, and the NLP was shown to be crystallized. Importantly, the peaks that corresponded to the peaks of PZQ were not noticed, which is an indication that PZQ interacted with NL and was well embedded in NL. Anti-calpain-engineered PZQ-loaded nanoliposomes were observed to be crystalline in nature with major peaks at 2θ-9.2°, 29.5°, and 42.6°, which could be another added advantage that there was a covalent attachment of the anti-calpain with the -OH group of DSPE-mPEG^2000^COOH during the formation of the anti-calpain-NLP.

#### 3.1.4. Thermal Behavior of the Formulated Nanoliposomes

**Figure 4:**
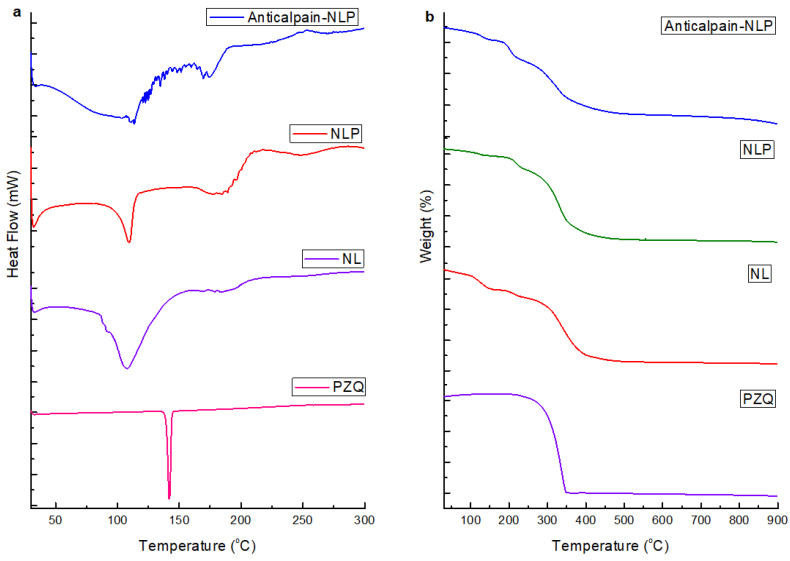
(**a**) DSC thermograms of PZQ, NL, NLP, and anti-calpain-NLP; (**b**) Thermogravimetric analyses of PZQ, NL, NLP and anti-calpain-NLP.

Differential scanning calorimetry (DSC) analysis was used to examine the formulated nanoliposomes’ thermal behavior to determine the changes in exothermic and endothermic temperature that happened during the nanoliposomes formulation. The data in Figure 4a showed the thermal curves of PZQ, NL, NLP, and anti-calpain-NLP. The endothermic peaks were observed at 137.99° for PZQ. The endothermic peaks for native nanoliposomes was observed at 111.09° and 198.22°, the NLP has endothermic peaks at 112.58° and 198.21°, and the endothermic peaks for the anti-calpain-NLPs were observed at 115.99° and 198.27°. The thermal behavior of the NLP showed that PZQ and the phospholipids of the nanoliposomes had a strong hydrophobic interaction during the entrapment of the drug. This was revealed by the lack of a peak corresponding to the peak of the drug for both the NLP and anti-calpain-NLP. The disappearance of the glass transition and the broadening of the endothermic peak in the anti-calpain-NLP suggested that there was a significant hydrophobic interaction between the PZQ and the antibody-functionalized nanoliposomes. Figure 4b demonstrated the thermal stability of the PZQ, NL, NLP, and anti-calpain-NLP using thermal gravimetric analysis. PZQ showed a major weight loss (95.4%), a temperature range of 270–342 °C, while both the native nanoliposomes and PZQ-loaded nanoliposomes and anti-calpain-functionalized PZQ-loaded nanoliposomes showed the same slow and steady weight loss pattern at similar temperature ranges. It can be deduced from this data that there is an improvement in the drug’s stability and showed the entrapment of the drug during the hydrophobic interaction between the PZQ and the phospholipids.

### 3.2. In Vitro Release Behavior of PZQ Analysis

**Figure 5:**
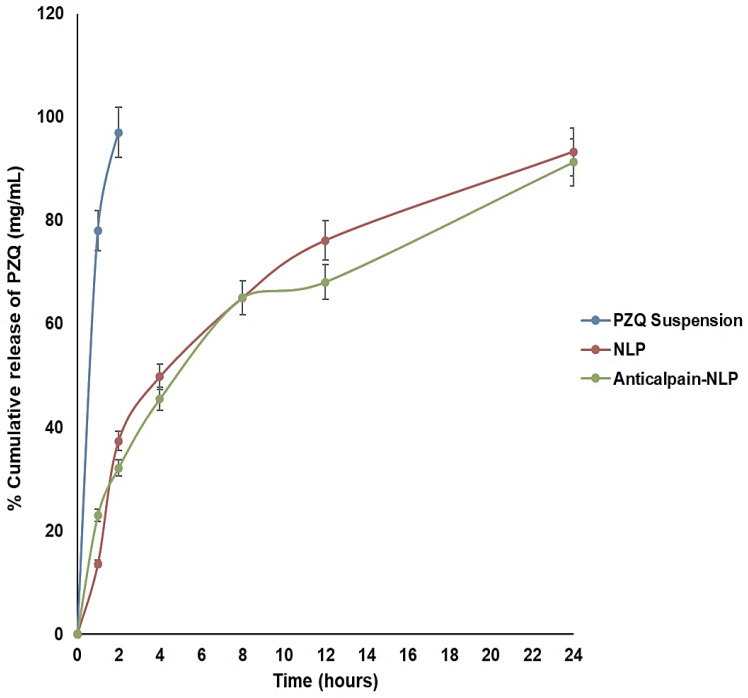
In vitro release profile of free PZQ and NLP. Release medium = PBS; Temperature = 37 ± 1 °C; pH = 7.4 (*n* = 3, mean ± SD).

The in vitro release ability of the PZQ-loaded nanoliposomes and PZQ-loaded functionalized nanoliposomes was evaluated in PBS (pH 7.4 at 37 °C) over 24 h. PBS (pH 7.4) was used to create pH conditions pertinent to the human physiological pH conditions and increase the analytical method’s sensitivity. Figure 5 demonstrates the percentage cumulative release profiles of PZQ, from the native PZQ (control), PZQ-loaded nanoliposomes, and PZQ-loaded functionalized nanoliposomes. Free PZQ suspension released virtually all free PZQ in 2 h, at 37 °C, under sink conditions. On the other hand, the formulated nanoliposomes showed an initial burst release pattern at 4 h, followed by approximately 93.2 and 91.1% of PZQ released within 24 h for PZQ-loaded nanoliposomes and PZQ-loaded functionalized nanoliposomes, respectively. Interestingly, both the PZQ-loaded nanoliposomes and PZQ-loaded functionalized nanoliposomes exhibited typical sustained release profiles. This sustained-release profile of PZQ exhibited by the formulated nanoliposomes may be attributed to the concentration of cholesterol, which is well-known to stabilize lipid bilayers through the reduction in membrane fluidity, thereby causing restriction in the movement of the drug across the formulated nanoliposomes. It could also be deduced that the rapid release of PZQ at the initial 4 h burst phase could be a result of the hydration process in the formulated nanoliposomes.

### 3.3. In Vitro Toxicity Analysis

**Figure 6:**
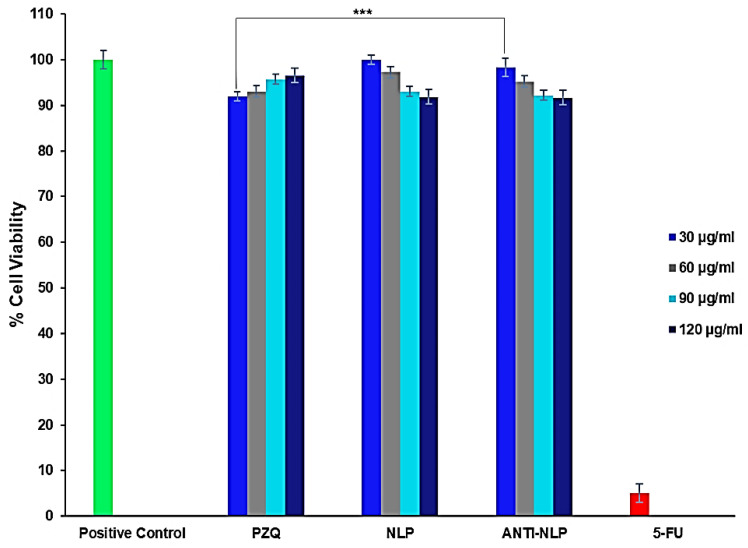
MTT assay measurement of the viability of RAW 264.7 murine macrophage cells after 24 h treatment with free PZQ, NLP, and anti-calpain-NLP at different concentrations (Data represent *n* = 3, mean ± SD). Positive control is the untreated group used to compute the percentage of cell viability. *** indicates *p* < 0.0001 when compared to the NLP and anti-calpain-NLP groups with the same concentration of PZQ.

**Figure 7:**
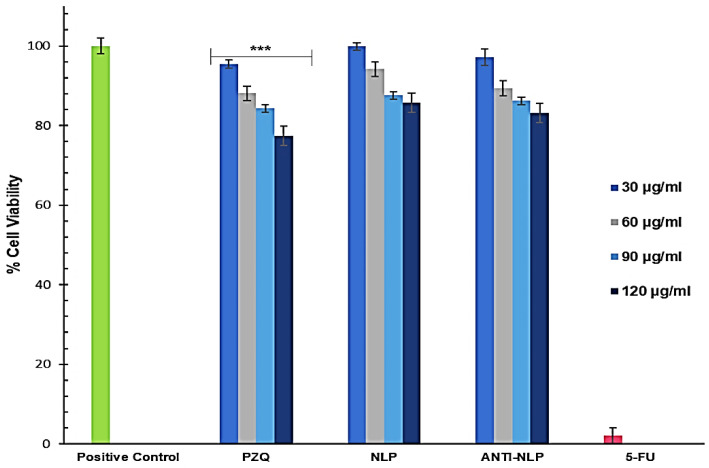
MTT assay measurement of the viability of 3T3 human fibroblast cells after 24 h treatment with free PZQ, NLP, and anti-calpain-NLP at different concentrations (data represent *n* = 3, mean ± SD). Positive control is the untreated group used to compute the percentage of cell viability. *** indicates *p* < 0.0001 when compared to the NLP and anti-calpain-NLP groups with the same concentration of PZQ.

The MTT (3-[4,5-dimethylthiazol-2yl]-2,5-diphenyl tetrazolium bromide) assay was used to investigate the cytotoxicity of the formulated nanoliposomes and PZQ on RAW 264.7 murine macrophage cells and 3T3 human fibroblast cells. **This MTT assay was used to measure the viability of RAW 264.7 murine macrophage and 3T3 human fibroblast cells after 48 h treatment with free PZQ, NLP, and anti-calpain-NLP at different concentrations, ranging from 30 to 120 μg/mL.** The results showed that the formulated nanoliposomes (NLP, and anti-calpain-NLP) and PZQ showed acceptable levels of cell viability, with no cytotoxic effects on the RAW 264.7 cells after 48 h and the viability is dose-dependent as shown in Figure 6. The opposite effect that was observed in the PZQ group, that is, an increase in cell viability with increasing concentration prompted the use of 3T3 human fibroblast cells for MTT assay to ascertain the cytotoxicity of the formulated nanoliposomes and PZQ. Thus, Figure 7 further showed that the concentrations ranging (from 30 to 120 μg/mL) employed in this study revealed acceptable levels of cell viability, with no significant cytotoxic effects on 3T3 human fibroblast cells. Interestingly, a significant increase (*p* < 0.0001) in the percentage of cell viability was observed in the PZQ tested group compared to the NLP and anti-NLP tested groups with higher viability. Moreover, it was revealed that the percentage of cell viability depends on the dose concentration of both the PZQ and the formulations.

### 3.4. Morphological Studies on RAW 264.7

**Figure 8:**
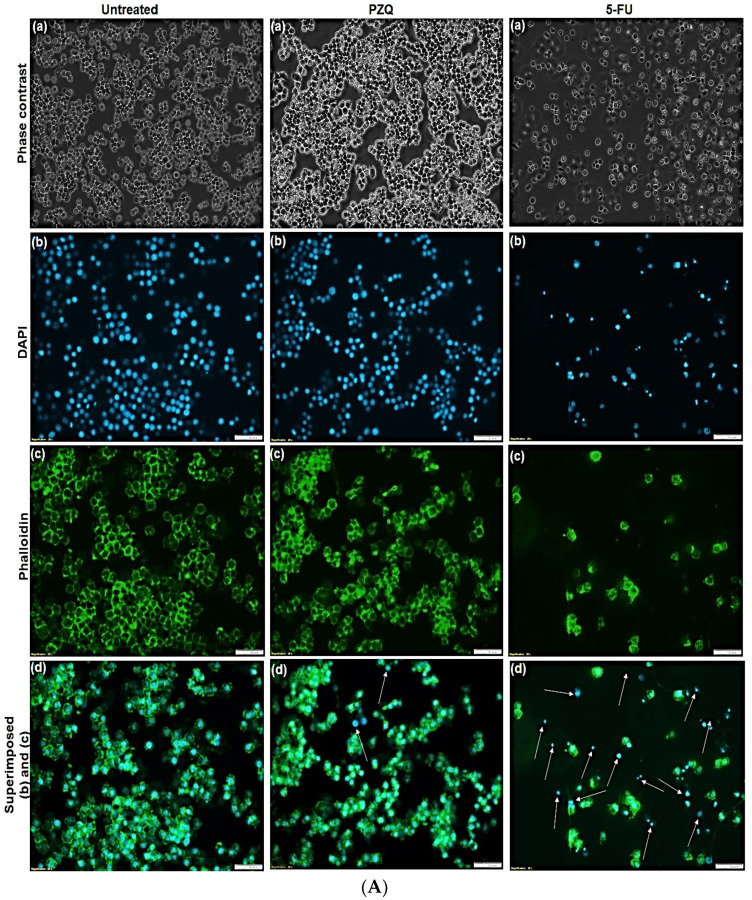

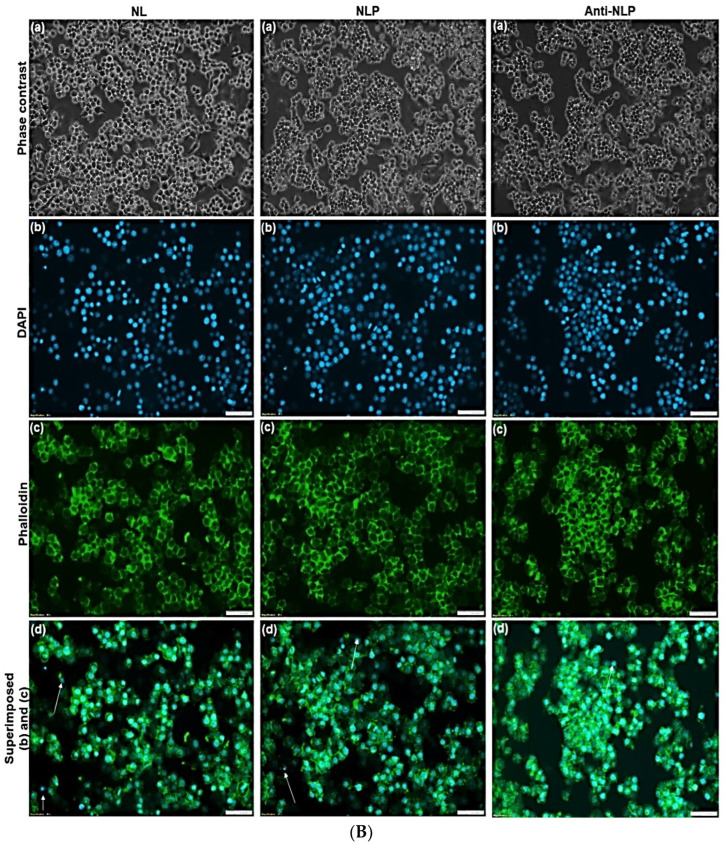
(**A**) Morphology analysis. Fluorescent microscopy images of RAW 264.7 macrophage cell (**a**) Phase contrast, (**b**) DAPI, (**c**) Phalloidin and (**d**) Superimposed of (**b**,**c**) for untreated (control), cell treated with 90 μg/mL of PZQ and 5-FU (10 μg/mL) (negative control). Scale bar: 100 μm; (×20 magnification). (**B**) Morphology analysis. Fluorescent microscopy images of RAW 264.7 macrophage cell (**a**) Phase contrast, (**b**) DAPI, (**c**) Phalloidin, and (**d**) Superimposed of (**b**,**c**) with 90 μg/mL of NL, NLP, and anti-calpain-NLP. Scale bar: 100 μm; (×20 magnification).

Figure 8A,B present the morphology of the RAW 264.7 murine macrophage cells visualized by fluorescent microscopy and inverted microscopy after the cells were treated with 90 μg/mL of free PZQ, NL, NLP, and anti-calpain-NLP and 5-FU (5-fluorouracil) (10 μg/mL) as the negative control, while using the untreated cells as the positive control. It was observed that all the cells treated with nanoliposomes and PZQ possess normal cell morphology of RAW 264.7 murine macrophage cells, and they were shown to have a round and smooth shape by phase-contrast images, while the cells treated with 10 μg/mL of 5-FU showed pseudopodia and cell shrinkage, which is the hallmark of apoptosis. Since DAPI staining can bind to DNA in the nucleus, while phalloidin binds to the F actin cytoskeleton of the cell membrane of the cells, they can thus be used to detect cells that have compromised membranes. In Figure 8A,B, the blue (DAPI) shows the nucleus of the cells, while the green (phalloidin) represent the cytoskeleton of the cells. It can be deduced by fluorescence microscopy that phalloidin did not stain the nucleus, as shown in the center of phalloidin images, which is the position of the nucleus. Furthermore, from the superimposition of the blue and green, it was revealed that the nanoliposomes did not show any direct cytotoxic effect on both the nucleus and the cytoplasm membrane of the RAW 264.7 murine macrophage cells after 24 h. Meanwhile, 10 μg/mL of 5-FU as a negative control ruptured the membrane and the cytoplasm of the cells, thereby exposing the nucleus.

**Figure 9:**
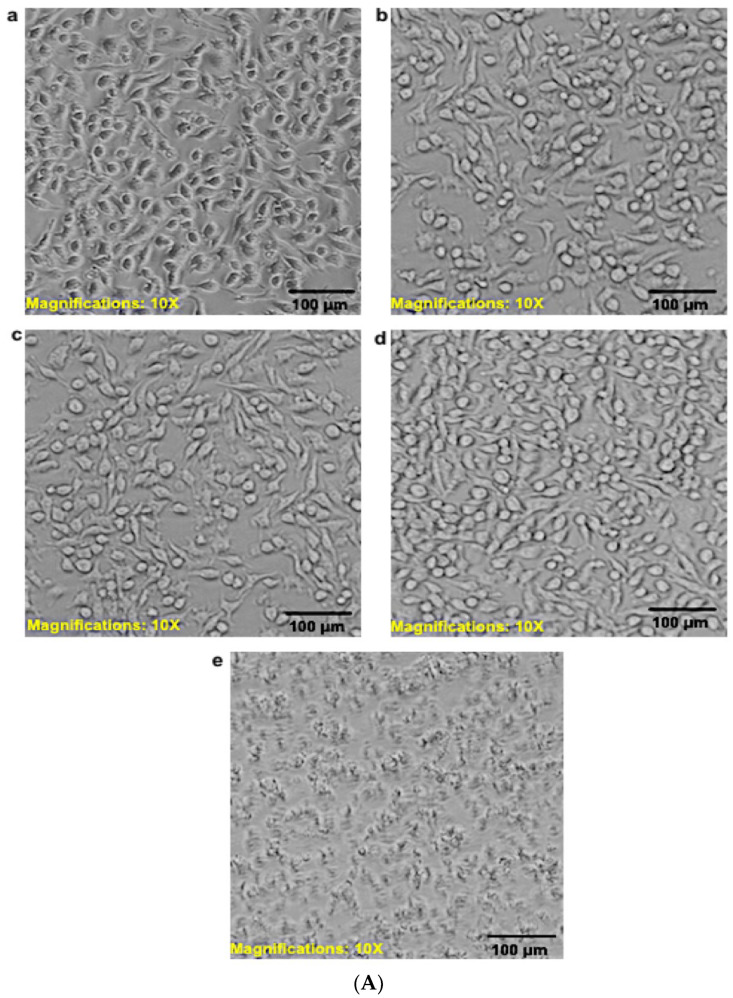

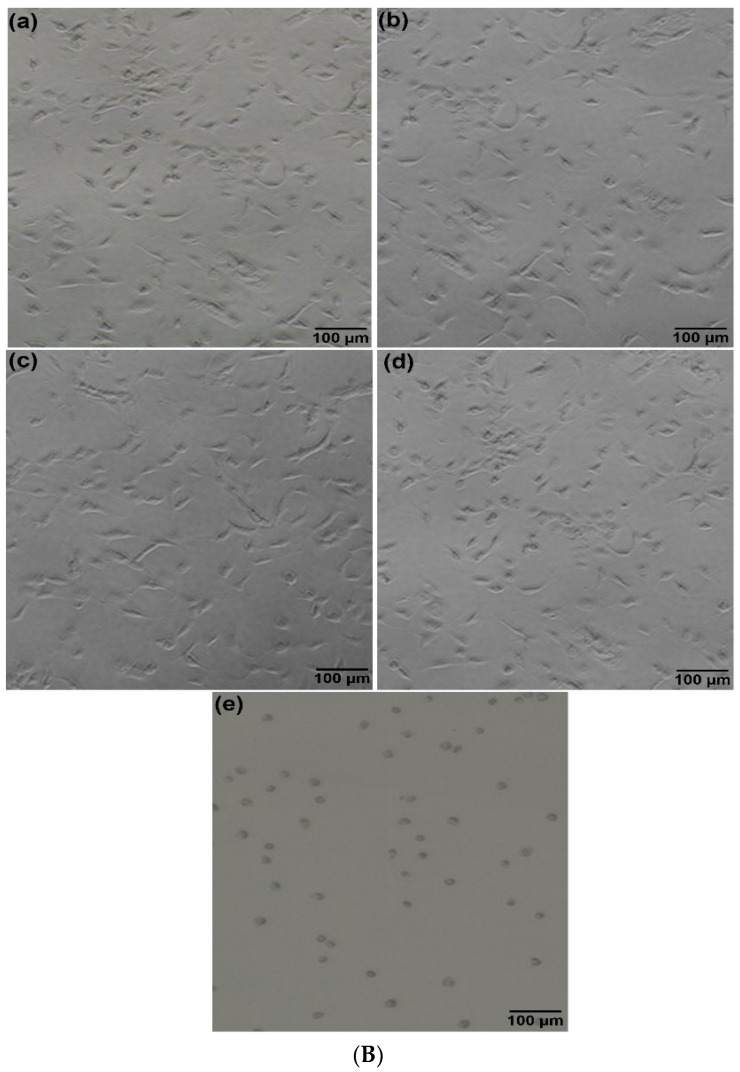
(**A**) Phase contrast morphology of RAW 264.7 murine macrophage cell visualized by inverted microscope (×10 magnification). The cells were treated with 30 μg/mL of Control (**a**), PZQ (**b**), NLP (**c**), anti-calpain-NLP (**d**), and 5-FU (**e**). (**B**) Phase contrast morphology of 3T3 human fibroblast cell visualized by inverted microscope (×10 magnification). The cells were treated with 30 μg/mL of Control (**a**), PZQ (**b**), NLP (**c**), anti-calpain-NLP (**d**), and 5-FU (**e**).

Figure 9A,B present the phase contrast morphology of the RAW 264.7 murine macrophage and 3T3 human fibroblast cells visualized by inverted microscopy after the cells were treated with 30 μg/mL of free PZQ, NLP, and anti-calpain-NLP and 10 μg/mL of 5-FU as negative control, while using the untreated cells as the positive control. It was observed that all the cells possessed normal cell morphology of RAW 264.7 murine macrophage and 3T3 human fibroblast cells, and they showed active structures due to the appearance of stellate and/or spindle shapes and centrally placed round nucleus or oval, as well as the abundant rough endoplasmic reticulum. However, the cells treated with 10 μg/mL of 5-FU (negative control) showed cell shrinkage, which resulted in apoptosis.

### 3.5. In Vivo Toxicity Analysis

**Figure 10:**
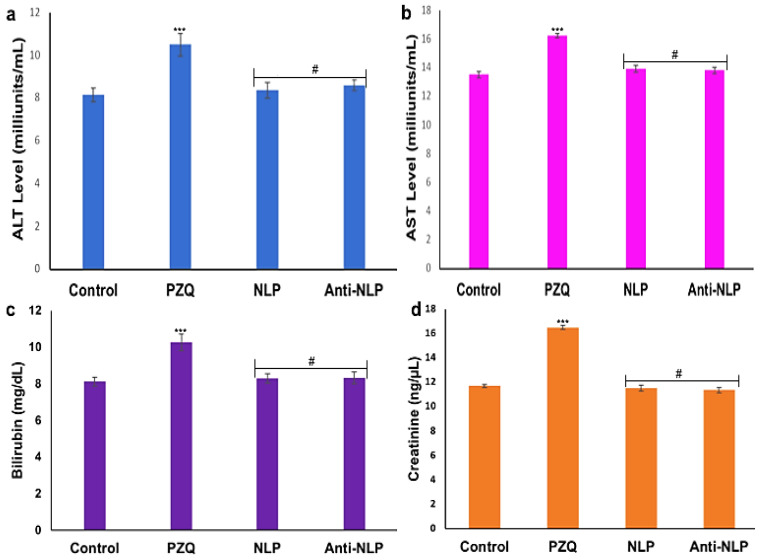
Biochemical markers (**a**) ALT (**b**) AST (**c**) Bilirubin and (**d**) creatinine levels in plasma. Values are expressed as mean ± standard deviation of five determinations (data represent *n* = 3, mean ± SD). *** indicates *p* < 0.0001 when compared to the control group, and # indicates *p* < 0.0001 when compared to the PZQ group.

The evaluation of different biochemical markers was carried out to ascertain the extent of potential damage to the kidney and liver. As shown in Figure 10, there is a significant increase (*p* < 0.0001) in the levels of biochemical markers (ALT, AST, creatinine, and bilirubin) in the plasma of the rats that received PZQ when compared to the control groups. It was further revealed that there is no significant increase in the biomarker levels of the groups that received the formulated nanoliposomes compared to the control. Interestingly, a significant decrease (*p* < 0.0001) was observed in the biochemical markers of the animals that received the formulated nanoliposomes compared with the PZQ groups. This indicates that the formulated nanoliposomes present no or minimal oxidative stress and confer hepatoprotective effects on the animals.

### 3.6. Histopathological Analysis

**Figure 11:**
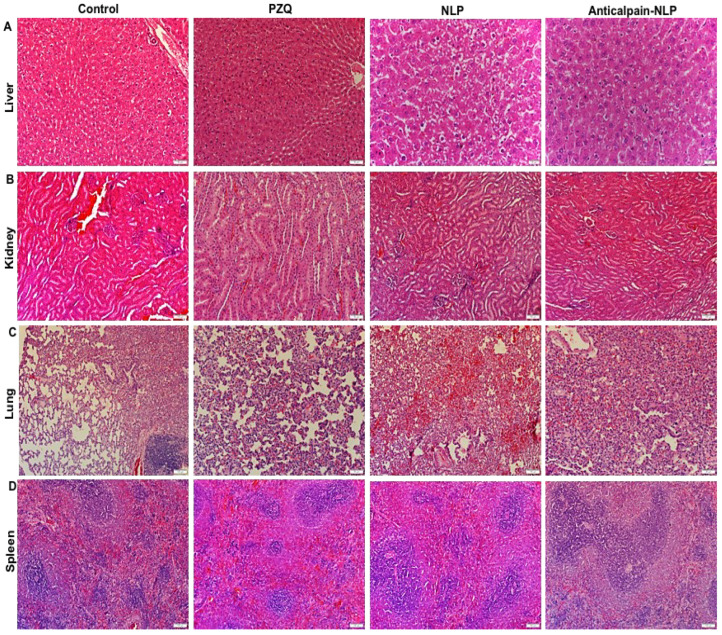
Histopathological sections of (**A**) liver, (**B**) kidney, (**C**) Lung, and (**D**) spleen after treating uninfected rats with 250 mg/kg of PZQ, NLP, and anti-calpain-NLP. Hematoxylin and eosin staining (×20 magnification).

Figure 11 depicts the histopathological examination images of untreated control, PZQ, NLP, and anti-calpain-NLP of the animal livers, kidneys, lungs, and spleens. A similar toxicity pattern was observed in histopathology sections of the liver, kidney, lung, and spleen. Hepatocyte granularity: This indicates active cellular function primarily due to swelling in cell organelles or increased cytoplasmic organelles. These organelles include smooth endoplasmic reticulum or peroxisomes, and mitochondria. It is thus not a pathological lesion, only indicative of increased cellular activity, which was present throughout the samples. Hepatocellular swelling and vacuolar change: The vacuolar difference is a non-specific indication of degeneration, and it is often considered borderline between adaptation, resolution, or inability to adapt. It can also be described as cytoplasmic alteration. In addition to degenerative changes developing due to hypoxic, toxic, metabolic, or inflammatory conditions, it may also occur due to glycogen accumulation. The latter may be of metabolic origin. Glycogen retention may also be a treatment-induced metabolic perturbation. The mild vacuolar change was present, mainly in the periportal regions. Renal tubular changes: The changes are minimal and only evident in a few tubules. They are likely hypoxic in origin, and thus probably terminal, primarily since the lesions are restricted to the deep cortical tissues where hypoxia would be first expected. However, further experiments are needed to confirm the hypothesis. Mild tubular epithelial cell swelling may develop due to disruption of ATP production, mitochondrial injury, free radical formation, peroxidation, or perturbed cell signaling. Pulmonary alveolar atelectasis: This finding correlated with the handling procedures and experimental design as blood collection from the heart may have caused pulmonary atelectasis. Terminal edema may develop, followed by euthanasia. Active bronchial-associated lymphoid tissue would be expected in animals exposed to respiratory pathogens and is an incidental finding. Normal active lymphoid tissue was shown in the spleen, with no observable abnormalities.

### 3.7. Assessment of Parasitological Cure Rate

**Figure 12:**
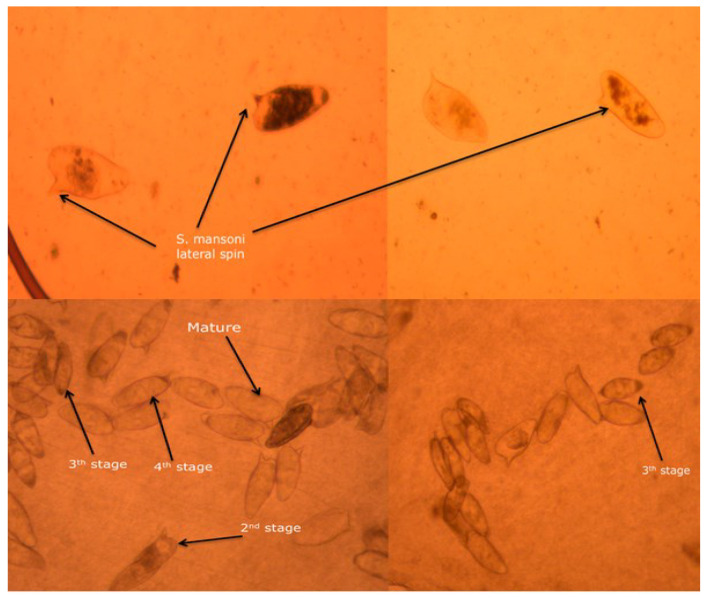
Typical imaging of *S. mansoni* eggs count at different stages of maturity in the small intestine of infected mice, visualized with the aid of a light microscope (×40 magnification).

**Figure 13:**
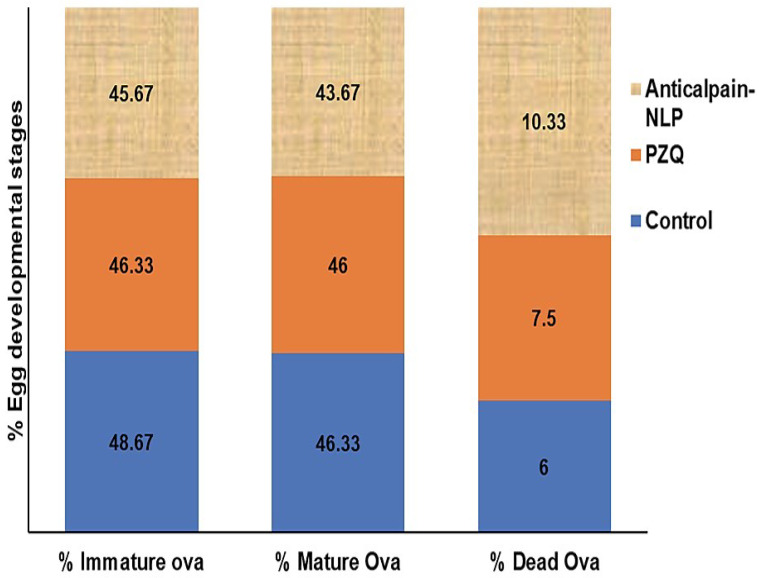
Effect of PZQ and anti-calpain-NLP (single dose 250 mg/kg two weeks post infection) on % egg developmental stages in *S. mansoni*-infected mice sacrificed six weeks post infection.

As shown in Figure 12, the imaging of how *S. mansoni* eggs were counted and the distribution and development of eggs according to their different maturity stage in the small intestine of infected mice are presented, which was similar to what has been reported previously in the literature by Mati and Melo [36]. Two weeks post-infection treatment data for worm recovery, oogram pattern, and egg count in tissues are shown in Table 1 and Table 2, and Figure 13. The oral administration of 250 mg/kg single dose of anti-calpain-NLP on worm load and sex in *S. mansoni*-infected mice resulted in a statistically significant percentage reduction in the total worm burden (Table 1) compared to the PZQ-treated group. As shown in Figure 13, there were statistically significant reductions in the percentage of immature and mature ova and an increase in the percentage of dead ova in the anti-calpain-NLP treated group compared to the control PZQ groups. Table 2 showed a statistically significant improvement in the percentage reduction in the ova count in the liver and intestine following the administration of 250 mg/kg single dose of PZQ equivalent in anti-calpain-NLP two weeks post infection compared to the PZQ and untreated control groups.

**Figure 14:**
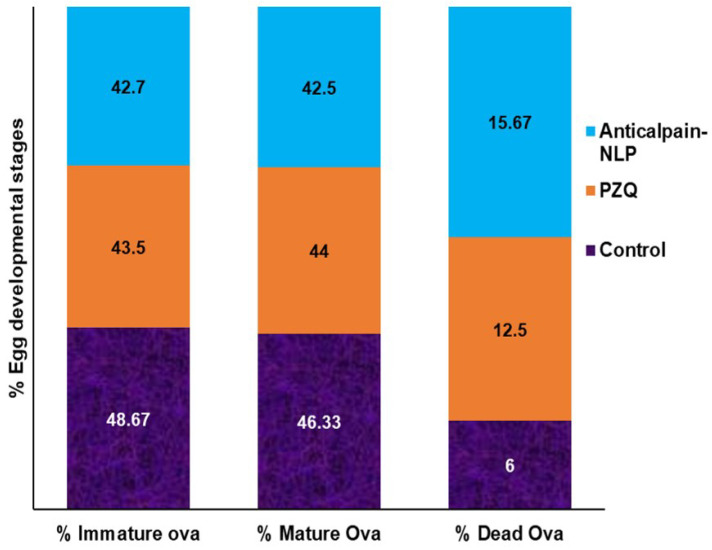
Effect of PZQ and anti-calpain-NLP (single dose 250 mg/kg four weeks post infection) on % Egg developmental stages in *S. mansoni*-infected mice sacrificed six weeks post infection.

Four weeks post-infection treatment data for worm recovery, oogram pattern, and egg count in tissues are shown in Table 3 and Table 4, and Figure 14 for adult worms. As shown in Table 3, there was a statistically significant reduction in the percentage of the total worm burden in the liver and porto-mesenteric of the anti-calpain-NLP compared to the PZQ-treated group. The oral administration of 250 mg/kg single dose of anti-calpain-NLP resulted in a statistically significant percentage of egg developmental stages in *S. mansoni*-infected mice compared to the infected untreated control and PZQ groups (Figure 14). However, improvement in the percentage reduction in the ova count in the intestine and liver was observed (Table 4) in an anti-calpain-NLP group compared to the PZQ and the infected untreated control groups.

## 4. Discussion

Praziquantel usage in the treatment of schistosome infections has been hampered by several issues, including low aqueous solubility, a shorter plasma half-life of roughly 1 to 2 h, and significant hepatic first-pass metabolism [15,16]. Other issues include praziquantel’s ineffectiveness against young schistosome worms, as well as drug tolerance and resistance in some parts of the world, which can be attributed to poor treatment compliance, parasite mutation rates, and overall parasite load, and co-infection with different Schistosoma parasite strains [14,18]. Thus, this work used nanotechnological approaches to design and construct liposomal nanosystems (LNS) and surface-functionalize the LNS with anti-calpain antibodies for targeted PZQ administration in schistosomiasis therapy.

Nanoliposomes have received a lot of interest in drug delivery because of their amphipathic nature, making them useful in enhancing and improving drug absorption across biological barriers, increasing the pace at which drugs may be released, and modifying drug solubility [15,37]. Due to considerable absorption by the reticuloendothelial system macrophage cells (RES), nanoliposomes, on the other hand, can decrease blood circulation times [38]. PZQ-loaded liposomes have been shown in several studies to improve PZQ anti-schistosomal activity, and this might be the basis for new PZQ administration routes [39,40]. Oral use of liposomes increases drug activity and bioavailability [41]. The fact that the liposome is a suitable carrier of drugs with poor water solubility, which is a feature of PZQ, might help explain the increase in availability. *S. mansoni* also possesses a phospholipid affinity, which might make the encapsulated drug more easily absorbed by the worm. When taken orally, the liposomal drug may shield the entrapped PZQ from digestive enzymes in the stomach, allowing the drug to reach the eggs and parasites in the liver.

In this study, the nanoliposomes synthesized are in the nano range with excellent polydispersity index and zeta potential. The average particle size and the PDI are some of the indications of the quality of the size distribution of nanocarriers [42,43]. The particle size distribution and PDI of lipid-based nanocarriers are essential physical properties to consider when dealing with pharmaceutical-grade products because the particle size affects encapsulation efficiency, stability, biodistribution, mucoadhesion, drug release profile, and cellular uptake [37,42,43,44]. It has been reported that particle size has a significant influence on drug delivery systems by influencing certain physiological processes such as tissue extravasation, tissue diffusion, hepatic uptake and accumulation (pharmacokinetics), and clearance through kidney excretion [43,45].

Moreover, the smaller the particle size nanocarriers (nanoliposomes), the larger the surface area and greater the capability to increase solubility, improve bioavailability, enable accurate targeting of the encapsulated drug, and enhance controlled release [37]. Additionally, nanocarriers with smaller particle sizes ranging from 20 to 100 nm may be easily distributed to the spleen, liver sinusoids, and bone marrow and may leave the bloodstream through the leaky capillaries of these organs to some extent [43]. Internal cellular binding and uptake of drug-loaded nanosystems are influenced by the nanoparticulate system’s shape and size [46,47]. Thus, the SEM and TEM images in this study revealed that the formulated nanoliposomes are in the nanosize range and uniformly spherical in shape with stable or intact structure; the SEM and TEM images were typical of the nanoliposomes [20,48] and corroborated the particle size, PDI and zeta potential results. It can be deduced from the physicochemical parameters (FTIR, DSC, XPDR, and TGA) data that there is an improvement in the drug’s stability and showed the entrapment of the drug during the hydrophobic interaction between the PZQ and the phospholipids.

Both the NLP and anti-calpain-NLP showed high PZQ entrapment and loading capacity, revealing that the nanoliposomes achieved high PZQ entrapment efficacy of 94.63 ± 0.30 and 92.07 ± 0.23, with a drug-loading capacity of 14.16 ± 0.61 and 9.03 ± 0.42, respectively. Other similar studies have reported the high encapsulation efficacy of lipophilic drugs in the lipid core of the lipid-based nanocarrier [39,49]. Moreover, nanoparticles have been investigated for precise targeting of brain tissues due to their small particle size and high drug encapsulation efficacy [50,51]. This showed that the formulated nanoliposomes could be used for the treatment of cerebral schistosomiasis. The formulated nanoliposomes showed an initial burst release pattern at 4 h followed by approximately 93.2 and 91.1% of PZQ released within 24 h for PZQ-loaded nanoliposomes and PZQ-loaded functionalized nanoliposomes, respectively. Interestingly, both the PZQ-loaded nanoliposomes and PZQ-loaded functionalized nanoliposomes exhibited typical sustained release profiles. The concentration of cholesterol, which is well known to stabilize lipid bilayers by reducing membrane fluidity, may be responsible for the prolonged release profiles of PZQ displayed by the formulated nanoliposomes, causing a restriction in the flow of drug across the formulated nanoliposomes [20,38]. It is also possible that the fast release of PZQ during the initial 4 h burst phase was caused by the hydration process in the formulated nanoliposomes.

A safety assessment of these nanoformulations in vitro revealed that the concentrations employed in this study, ranging from 30 to 120 μg/mL, were at acceptable levels of cell viability, with no significant cytotoxic effects on RAW 264.7 murine macrophage cells and 3T3 human fibroblast cells. **Moreover, it was revealed that the percentage of cell viability depends on the dose concentration of both the PZQ and the lipoidal nanosystem formulations (NLP and Anti-NLP).** Interestingly, a significant increase (*p* < 0.0001) in the percentage of cell viability was observed in the PZQ group compared to the NLP and anti-NLP groups. This in vitro cytotoxicity cell culture result was further supported by in vivo toxicity studies where the extent of PZQ, NLP, and anti-calpain-NLP was shown in the histopathological analysis of different organs and evaluation of biochemical markers. Based on these observations, the fabrication of NLP and engineering of NLP with anti-calpain antibody play an indispensable role in circumventing the traces of the toxic aspects associated with the drug.

Additionally, the extent of safety of the formulated nanoliposomes was shown, and the biochemical markers and histopathological examinations present no or minimal oxidative stress and confer hepatoprotective effects on the animals. **However, an oxidative stress marker evaluation is needed to confirm this observation.** In severe instances of intestinal schistosomiasis, liver enlargement is prevalent, and it is often accompanied by a buildup of fluid in the peritoneal cavity and abdominal blood vessel hypertension. Spleen hypertrophy is also possible in such circumstances [52,53]. Moreover, hematuria is the most common symptom of urogenital schistosomiasis (blood in urine) [53]. In advanced cases, fibrosis of the bladder and ureter, as well as kidney impairment, may be discovered [53]. Thus, the anti-calpain-NLP would localize, recognize and bind to the schistosomes without posing any adverse effects on the organs or tissues.

The cure rate of the anti-calpain-NLP and PZQ was assessed by parasitological analysis, and it was discovered that the treatment with 250 mg/kg of drug equivalent in anti-calpain-NLP showed more significant activity on the total worm burden, ova count in both the intestine and the liver. Additionally, the anti-calpain-engineered lipoidal nanosystem, when compared with the same dose of PZQ, showed a greater effect on both the mature and immature ova, as well as caused ova dead during the egg developmental stages in both the young and adult worm represented by two weeks and four weeks post-infected mice, respectively. The calculation of the reduction in the total worm burden, egg developmental stages, the number of eggs/gram tissue and the percentage of reduction in total ova/gram tissue is the criteria for parasitological cure rate [33,34,35]. These enormous effects of the anti-calpain-engineered nanoliposomes on schistosomes eggs, and worms may be due to the target rate and greater absorption of the nanosystem by the worms and the eggs in the liver, porto-mesenteric, and intestine. The effects of the anti-calpain surface engineering of the lipoidal nanosystem could be due to the suppression of the calpain protein activity. This could lead to the blocking of cell–cell adhesion or cell–substrate adhesion, which occur via the host–parasite interfaces, impair the schistosomes’ capacity to conduct calcium-mediated signaling, and stop surface membrane biogenesis, i.e., the surface membrane synthetic process. Since the calpain host–parasite interface is crucial for the schistosome’s developmental activities, failure in the supply and transfer of membrane precursors might cause the tegument to rupture, disrupting the worm’s capacity to maintain a solute balance [26,27].

## 5. Conclusions

**The findings of this study revealed that anti-calpain-functionalized nanoliposomes might be used to improve the transport of PZQ into the liver and intestines for targeting both the young and adult schistosomes for schistosomiasis treatment.** It was discovered that the nanoliposomes synthesized are in the nanoscale range with excellent polydispersity index and zeta potential. The SEM and TEM images in this study revealed that the formulated nanoliposomes are in the nanosize range and uniformly spherical with stable or intact structure, with typical SEM and TEM images of the nanoliposomes corroborating the particle size, PDI, and zeta potential results. Moreover, the physicochemical parameters (FTIR, DSC, XPDR, and TGA) data revealed that there is an improvement in the stability of the drug and also showed the entrapment of the drug during the hydrophobic interaction between the PZQ and the phospholipids. It was shown that both the NLP and anti-calpain-NLP showed high PZQ entrapment and loading capacity. Interestingly, both the PZQ-loaded nanoliposomes and PZQ-loaded functionalized nanoliposomes exhibited typical sustained release profiles. An in vitro safety assessment of these nanoformulations revealed that the concentrations employed in this study, ranging from 30 to 120 μg/mL, revealed acceptable levels of cell viability, with no significant cytotoxic effects on RAW 264.7 murine macrophage and 3T3 human fibroblast cells. The extent of safety of the formulated nanoliposomes was shown and the biochemical markers and histopathological examinations present no or minimal oxidative stress and confer hepatoprotective effects on the animals. In *S. mansoni*-infected mice, a single 250 mg/kg oral dosage of drug equivalent in anti-calpain-NLP significantly increased PZQ antischistosomal activity compared to free PZQ. The findings obtained support the ability of oral anti-calpain-NLP to target young and adult schistosomes in the liver and porto-mesenteric locations, resulting in improved effectiveness of PZQ, hence being a promising therapeutic strategy against schistosomiasis.

## Figures and Tables

**Table 1:** Effect of PZQ and anti-calpain-NLP (single dose 250 mg/kg two weeks post infection) on worm load and sex in *S. mansoni*-infected mice sacrificed six weeks post infection.

	Mean Worm Burden ±SD				% Reduction in Total Worm Burden
	(Liver and Porto-Mesenteric)				
	Male	Female	Couples	Total	
Control	2.33 ± 0.81	0.33 ± 0.52	6.17 ± 0.75	15 ± 0.89	
PZQ	1.33 ± 1.21	0	4.33 ± 0.82	10.0 ± 1.41	33.3
Antical-NLP	1.10 ± 0.52	0.17 ± 0.41	3.67 ± 1.37	6.83 ± 2.4	54.5

**Table 2:** Effect of PZQ and anti-calpain-NLP (single dose 250 mg/kg two weeks post infection) on number of ova/gm tissues in *S. mansoni*-infected mice sacrificed six weeks post infection.

Mice Group	Liver	% Reduction in Ova Count in Liver	Intestine	% Reduction in Ova Count in the Intestine
Control	28,202 ± 4372		31,902 ± 4342	
PZQ	20,303 ± 2175	28.00	22,702 ± 5347	28.84
Antical-NLP	12,387 ± 2951	56.07	13,436 ± 2332	57.88

**Table 3:** Effect of PZQ and anti-calpain-NLP (single dose 250 mg/kg four weeks post infection) on worm load and sex in *S. mansoni*-infected mice sacrificed six weeks post infection.

	Mean Worm Burden ±SD				% Reduction in Total Worm Burden
	(Liver and Porto-Mesenteric)				
	Male	Female	Couples	Total	
Control	2.33 ± 0.81	0.33 ± 0.52	6.17 ± 0.75	15.00 ± 0.89	
PZQ	1.50 ± 1.40	0	2.00 ± 0.89	5.50 ± 2.60	63.3
Antical-NLP	1.00 ± 0.63	0	1.50 ± 0.83	4.17 ± 1.47	72.2

**Table 4:** Effect of PZQ and anti-calpain-NLP (single dose 250 mg/kg four weeks post infection) on number of ova/gm tissues in *S. mansoni*-infected mice sacrificed six weeks post infection.

Mice Group	Liver	% Reduction in Ova Count in Liver	Intestine	% Reduction in Ova Count in the Intestine
Control	28,202 ± 4372		31,902 ± 4342	
PZQ	13,626 ± 2936	51.68	14,658 ± 3699	54.05
Antical-NLP	10,112 ± 3745	64.14	9310 ± 3789	70.81
